# Improved fidelity of orientation perception: a learning effect dissociable from enhanced discriminability

**DOI:** 10.1038/s41598-020-62882-3

**Published:** 2020-04-20

**Authors:** En Zhang, Wu Li

**Affiliations:** State Key Laboratory of Cognitive Neuroscience and Learning & IDG/McGovern Institute for Brain Research, Beijing Normal University, Beijing, 100875 China

**Keywords:** Visual system, Human behaviour

## Abstract

Visual perception can be influenced by stimulus context, selective attention, and prior experience. Many previous studies have shown complex interactions among these influencing factors, but it remains unclear whether context-induced illusions could be reduced by perceptual training and whether such a change in perceptual fidelity is linked to improved perceptual discriminability. To address this question, we introduced a context-induced tilt illusion into an orientation discrimination training paradigm. This resulted in parallel and long-term improvements in the discriminability and fidelity of orientation perception. The improved discriminability was specific to the task-relevant target stimulus but nonspecific to the task-irrelevant context. By contrast, the improved perceptual fidelity was specific to the task-irrelevant contextual stimulus that induced the illusion, but not specific to the task-relevant target stimulus or task performed on one of its features. These results indicate two dissociable learning effects associated with the same training procedure. Such a dissociation was further supported by the observation that the sizes of the two learning effects were uncorrelated across the subjects. Our findings suggest two parallel learning processes: a task-dependent process giving rise to enhanced discriminability for the task-relevant stimulus attribute, and a context-dependent process leading to improved perceptual fidelity for the attended stimuli.

## Introduction

The brain possesses remarkable perceptual abilities, such as to discriminate a small difference in orientation between two lines and to detect a visual target camouflaged within distractors. A large number of studies have demonstrated that such superior abilities can be further improved with training and that the practice effects are usually specific to the trained stimulus and task settings (see reviews^1,2^).

Visual feature discriminability is only one aspect of perceptual ability. Another important aspect is perceptual fidelity, namely the faithfulness of our subjective percept to the objective reality. Perceptual discriminability and fidelity, and visual perception in general, can be affected by both external and internal factors.

An important external influencing factor is the context within which a visual item is displayed. Visual scene components usually form complex spatial and temporal context, which can interfere with the fidelity of the perceived stimulus. For example, vertical lines appear tilted when they are surrounded by oblique lines (known as the tilt illusion) or when their occurrence is preceded by an exposure to oblique lines (known as the tilt aftereffect) (see review^3^). Contextual influences on visual perception are closely correlated with contextual modulation of visual processing (see reviews^3,4^). When a visual stimulus is placed within the classical receptive field of a neuron in the visual cortex, responses of the neuron are profoundly modulated by surrounding stimulus context (for example see ref. ^5–8^). Contextual influences seen in the primary visual cortex (V1) are thought to be mediated by long-range horizontal connections within V1 and feedback connections from higher cortical areas^9–15^. Compared with the conventional tuning properties of V1 neurons, contextual modulations in V1 show similar but broader selectivities for stimulus features such as orientation, spatial frequency and motion speed^8^. Orientation-dependent contextual modulation in V1 could be the neural substrate of the tilt illusion^16^.

An important internal factor influencing visual processing and perception is top-down attention. Of particular interest is the interplay among attention, contextual influences, and experience or training. Some studies have explored, in the presence of visual illusions induced by spatial or temporal context, experience-dependent changes in perceptual fidelity. It has been reported that prolonged (hours or days) adaptation to the same visual stimuli (i.e. adapters) can induce long-lasting changes in perceptual fidelity, such as decreased visual aftereffects in perception of forms^17^, luminance contrast^18^, and motion speed^18,19^. These adaptive changes are affected by attention (or the task-relevancy of the adapters) during repeated adaptation^18,19^. In addition to the temporal context and aftereffects, the influences of spatial context can also be reduced by training on attentional deployment^20,21^. Accumulated evidence from perceptual learning studies supports that training can cause multiple changes, including refined sensory representation, attentional control and decision making^2,22^.

Several studies have also explored the changes in perceptual fidelity induced by perceptual training in the absence of stimulus context^23,24^. After training human observers to discriminate the orientation of an isolated grating patch, one study reported the emergence of an aftereffect-like bias in orientation perception^23^, which is predictable according to a computational modeling study^25^; another study examined the post-training tilt illusion at the trained orientation and did not see any noticeable difference from the pre-training illusion, suggesting that perceptual discriminability and fidelity are not inherently linked^24^.

The tilt illusion is a paradigmatic example of contextual influences on perceptual fidelity; however, to our knowledge, no studies have explored whether and how perceptual fidelity and discriminability can be altered by training in the presence of the tilt illusion. In particular, it remains unknown as to whether the perceptual bias reflected in the tilt illusion can be reduced in a long-term fashion by perceptual training, and whether such a change in perceptual fidelity is linked to the improvement in perceptual discriminability. Answers to these questions are important to further our understanding of the interplay between contextual influences, attentional modulation and perceptual learning in shaping our perception.

In the current study, human subjects were trained, in the presence of the tilt illusion, to discriminate the orientations of grating stimuli. The experimental design allowed us to simultaneously measure the discriminability and fidelity of orientation perception over the course of training and to dissect the properties of the learning effects on these two perceptual abilities.

## Methods

### Subjects

Naive subjects with normal or corrected-to-normal eyesight were recruited from undergraduate and graduate students. Each subject was required to sign an informed consent form and was unaware of the purpose of the study. The current study conformed to the Declaration of Helsinki and was approved by the ethics committee of Beijing Normal University.

### Visual stimuli

The visual stimuli were displayed on a CRT monitor (Iiyama Vision Master Pro-514, 100 Hz refresh rate, 1200 × 900 resolution, 120 cm viewing distance) by using a stimulus generator (ViSaGe, Cambridge Research Systems). A head-and-chin rest was used to stabilize the subject’s head. All experiments were conducted in a dimly lit room.

**Figure 1:**
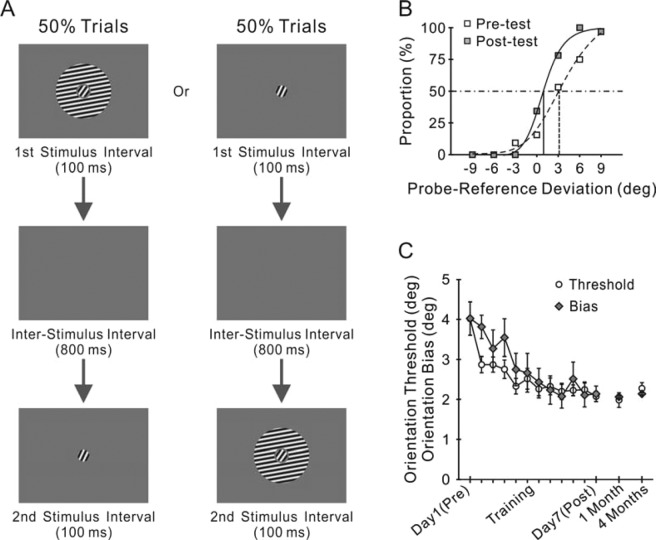
Two parallel practice effects of orientation discrimination training. (**A**) Visual stimuli in a trial. The probe grating patch could be displayed in either the second (left) or the first (right) stimulus interval at an equal probability of 50% within a block of trials. (**B**) Examples of the pre- and post-test psychometric curves from a subject. Each data point was based on 32 trials, showing the proportion of trials in which the subject reported the probe gratings (without the surround) as tilted clockwise relative to the reference (with the surround) at the specified orientation deviation between them. The orientation discrimination threshold was measured as half of the difference in probe-reference deviation between the 25% and the 75% performance levels according to the fitted psychometric curve. The perceptual bias (i.e. the tilt illusion) was calculated from the point of subjective equality (the intersection between the horizontal line and the psychometric curve), at which the observer perceived no difference in orientation between the probe and reference gratings. (**C**) Learning curves averaged across the subjects (n = 10), showing a progressive reduction in both the discrimination threshold and perceptual bias. The four isolated data points indicate the values tested again after one and four months. Error bars indicate ±SEM.

On each trial the subjects first gazed at the screen center for 600 ms. Subsequently, two stimulus patterns were presented at the screen center in a random sequence within two stimulus intervals (Fig. 1A), each for 100 ms with an inter-stimulus interval of 800 ms. One pattern was formed by a circular patch of gratings (referred to as the reference) surrounded by an annular patch of gratings (referred to as the inducer, which was used to generate the tilt illusion on the reference); the other pattern (referred to as the probe) was similar to the reference gratings except for a small difference in orientation or size. The gratings were phase-randomized, stationary, sinusoidal with 80% Michelson contrast, 3 cycle/° spatial frequency, and 0.50 cd/m2 mean luminance that was equal to the screen background. The low mean luminance was used to minimize the visibility of screen borders that might interfere with orientation perception of the stimuli.

### Behavioral tasks

The current study involved the following three discrimination tasks performed on the stimuli described above.

In the orientation discrimination task, the reference and the probe stimuli were 1° in diameter; the inducer was 1.2° and 4° in inner and outer diameters. Within a block of trials, the probe gratings were randomly set at one of seven orientations, which was 0, 3, 6 or 9 degrees clockwise or counterclockwise deviated from the fixed orientation of the reference gratings. The subjects were required to ignore the surround inducer and report, by pressing one of two keys, whether the small circular grating patch in the second stimulus interval was tilted clockwise or counterclockwise relative to that in the first interval (Fig. 1A). This design allowed us to simultaneously measure the discrimination threshold and the tilt illusion (i.e. the perceptual bias) under the same experimental condition (see *Data analyses*).

In the size discrimination task, the orientation was always identical for the reference and probe gratings. The diameter of the probe was randomly selected from seven pre-defined values, which were 0, 1.8, 3.6 or 5.4 arc minutes (corresponding to 0, 2, 4, 6 pixels, respectively, at the viewing distance used) larger or smaller than the reference. The subjects were required to report whether the circular grating patch in the second stimulus interval was larger or smaller relative to that in the first interval. The size discrimination was used for examining whether the orientation illusion could be affected by training in a task irrelevant to orientation perception.

In the luminance discrimination task, the reference and probe grating patches were identical, each with an additional dot (0.2° in diameter) embedded in the center. The dot on the reference gratings was set to the mean luminance of gratings (0.50 cd/m2); the luminance of the dot on the probe gratings differed by 0, ±0.1, ±0.2 or ±0.3 cd/m2. The subjects were instructed to report whether the dot in the second stimulus interval was brighter or dimmer than that in the first interval. This task allowed us to examine whether passive exposure to the stimulus display would affect the orientation tilt illusion.

No feedback was given on the observer’s responses because external feedback signals could confound the assessment of the tilt illusion. To minimize the influences of procedural learning, the naive subjects were given several tens of practice trials to get familiar with the behavioral tasks before data collection.

### Experiment 1

Ten naive subjects were trained in the orientation discrimination task in which the center reference gratings were fixed at 55° orientation and the surround inducer gratings at 10° (referred to as the C55/S10 condition; similar denotation was used for other Center/Surround stimulus configurations). The training and testing procedures were divided into blocks of 56 trials (8 trials for each of the 7 probe orientations). The pre- and post-training thresholds were measured using 4 blocks of trials on day 1 and 7, respectively, between which the subjects were trained each day for 12 blocks of trials (a total of 672 trials per day). The subjects were called back one and four months later for testing whether the learning effects were retained.

### Experiment 2

**Figure 2:**
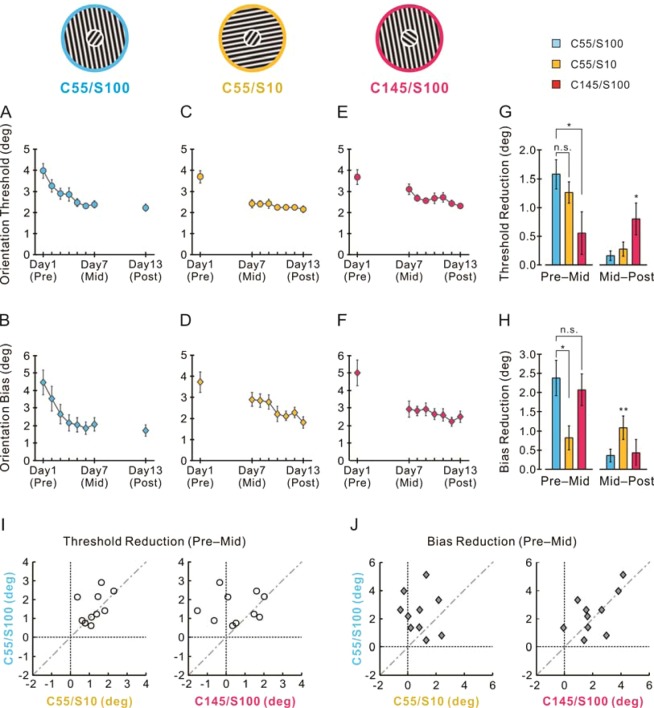
Orientation specificities of the learning effects. Averaged orientation discrimination threshold (**A**,**C**,**E**) and perceptual bias (**B**,**D**,**F**) (n = 10 subjects) are plotted as a function of the training days for the three stimulus conditions (top insets). Between the pre- and mid-tests, only the C55/S100 was trained for five days. After this first training stage and the mid-test, the C55/S10 (center rotated) and C145/S100 (surround rotated) were trained together for another five days (day 8–12). Based on the data presented in (**A**–**F**), we compared the average reductions in the orientation threshold (**G**) and perceptual bias (**H**) for the three center-surround stimulus configurations in the first (left panel) and second (right panel) training stages. Data from individual subjects contributing to (**G**,**H)** are shown in (**I**,**J**) respectively. All error bars indicate ± SEM.*p < 0.05; **p < 0.01.

Another ten naive subjects performed the orientation discrimination task using three center-surround configurations, C55/S100, C55/S10 and C145/S100, in which the orientation contrast between the center reference and surround inducer was kept identical at 45° (Fig. 2 insets). After the pre-test (day 1; 4 blocks of 56 trials for each of the three configurations), the subjects were trained in the C55/S100 condition from day 2 to 6 (the first training stage, 12 blocks per day). On day 7 (mid-test) we changed the reference and inducer orientations independently. We measured whether the illusion and threshold reductions in the trained C55/S100 condition could transfer to the two untrained conditions of C55/S10 (with rotated surround) and C145/S100 (with rotated center), using 4 blocks of trials for both conditions. To further verify the transferability or specificity of the practice effects, the two untrained conditions were subsequently trained for 5 days (the second training stage, 6 blocks per day for each condition).

### Experiment 3

Six naive subjects were trained in the size discrimination task using the stimulus settings similar to those in Experiment 1 (the C55/S10 condition, with the probe gratings fixed at the 55° reference orientation). In this experiment, the pre-training orientation discrimination threshold and perceptual bias were not tested to avoid the learning effects induced by the pre-test itself. The subjects performed the size discrimination task from day 1 to 6 (the first training stage), 4 blocks of trials in the first day and 12 blocks in the following days. From day 7 to day 13 (the second training stage), the subjects underwent the same training process of orientation discrimination as in Experiment 1. By measuring the practice effects in the second training stage, we were able to infer to what extent the orientation threshold and illusion were affected by the prior size discrimination training, which was irrelevant to orientation perception.

### Experiment 4

Five naive subjects were first trained in the luminance discrimination task by simply attending to the luminance change of the dots in the two stimulus intervals. The stimulus settings were similar to Experiment 1 and 3 (the C55/S10 condition, with the probe gratings fixed at the 55° reference orientation). The subjects performed the dot luminance task from day 1 to 6 (the first training stage), 4 blocks of trials in the first day and 12 blocks in the following days. From day 7 to day 13 (the second training stage), the subjects underwent the same training process of orientation discrimination as in Experiment 1 and 3. By measuring the practice effects in the second training stage, we were able to infer to what extent passive exposure to the grating patches in the first training stage affected the orientation threshold and illusion.

### Data analyses

Based on data collected in the orientation discrimination task, we calculated, for each of the seven orientation deviations between the probe and reference gratings, the proportion of observers’ responses in which the probe gratings were reported as clockwise tilted relative to the reference. Note that the probe could be presented randomly in either the first or the second stimulus interval in a block of trials (Fig. 1A). A psychometric curve was constructed using the seven response ratios (Fig. 1B). By fitting the psychometric curve with a logistic function, we defined the orientation discrimination threshold (i.e. discriminability) as one half of the horizontal distance between the 75% and 25% data points^26^. The thresholds for size and luminance discrimination in Experiment 3 and 4 were calculated similarly. We also measured, on the same psychometric curve, the context-induced bias in orientation perception (i.e. the tilt illusion) as the probe-reference orientation deviation at which the observers’ response was 50% (Fig. 1B).

Two-tailed paired t-test was used to examine whether the learning effects averaged across the subjects were statistically significant. To test for statistical significance of learning transferability among the three center-surround stimulus configurations in Experiment 2, we performed ANOVA followed by post-hoc Tukey’s HSD test for multiple comparisons.

## Results

### Two parallel learning effects associated with orientation discrimination training

We first investigated in Experiment 1 whether orientation discrimination training in the presence of the tilt illusion could affect perceptual fidelity. We used the C55/S10 center-surround configuration (reference gratings at 55° orientation, surround inducer at 10°; Fig. 1A). This configuration defined a 45° center-surround orientation contrast that is known to produce the repulsion form of tilt illusion (the central gratings appear tilted further away from the surround gratings as compared to its actual orientation)^26^. At larger center-surround orientation contrasts the tilt illusion can take the form of attraction (the central gratings appear tilted closer to the surround gratings), but the attractive effect is very weak (see review^3^). Therefore, the much stronger repulsion illusion was used in the current study.

Ten naive subjects were trained to discriminate the orientation differences between the reference and the probe gratings (Fig. 1A). Expectedly, training resulted in a significant decrement in discrimination threshold (Fig. 1C; pre-test 4.02 ± 0.42° vs. post-test 2.07 ± 0.13°, n = 10, t = 4.20, p = 0.002, two-sided paired t-test). The perceptual bias was also reduced in parallel (Fig. 1C; pre-test 4.02 ± 0.42° vs. post-test 2.14 ± 0.20°, n = 10, t = 3.48, p = 0.007).

To examine whether the two learning effects were long lasting, the subjects were tested again after one and four months. The reductions in both the threshold and the bias were retained (Fig. 1C; post-test threshold 2.07 ± 0.13° vs. four months later 2.28 ± 0.21°, n = 10, t = 1.36, p = 0.21; post-test bias 2.14 ± 0.20° vs. four months later 2.14 ± 0.32°, n = 10, t = 0.00, p = 1.00).

Experiment 1 showed that orientation discrimination training in the presence of the tilt illusion resulted in two parallel and long-lasting learning effects on perception of the task-relevant stimulus, enhancing the orientation discriminability (as seen in the reduced discrimination threshold) and increasing the fidelity of orientation perception (as seen in the reduced perceptual bias).

### Orientation specificities of the two learning effects

Orientation discrimination learning is known to be specific to the trained stimulus orientation. In Experiment 2 we examined the orientation specificity and transferability of the two learning effects. We changed the orientation of either the center reference or the surround inducer but kept their orientation contrast at 45°. Another ten naive subjects performed the orientation discrimination task using the three center-surround stimulus configurations (C55/S100, C55/S10 and C145/S100; Fig. 2 insets) on day 1 (pre-test), day 7 (mid-test) and day 13 (post-test). For these stimulus configurations, the pre-test orientation thresholds (Fig. 2A,C,E) and the pre-test orientation bias (Fig. 2B,D,F) were comparable for the chosen stimulus settings, allowing direct comparisons of the raw values across different conditions.

After the pre-test of all three configurations on day 1, only the C55/S100 was trained for five days (from day 2 to 6). Similar to Experiment 1, training decreased the orientation threshold (Fig. 2A, pre-test 3.96 ± 0.33° vs. mid-test 2.39 ± 0.17°, n = 10, t = 6.27, p = 0.0002, two-sided paired t-test) and the orientation bias (Fig. 2B, pre-test 4.45 ± 0.72° vs. mid-test 2.08 ± 0.38°, n = 10, t = 5.14, p = 0.0006).

In order to examine the specificity or transferability of the two learning effects, we compared the learning-induced changes in orientation threshold and bias, respectively, between the trained (C55/S100) and untrained (C55/S10, C145/S100) configurations. One-way ANOVA showed that the stimulus configuration was a significant influencing factor for the observed practice effects (for the threshold decrements F(2, 27) = 3.479, p = 0.045; for the bias decrements F(2, 27) = 4.181, p = 0.026).

We used the Tukey’s HSD test for post-hoc multiple comparisons. With respect to the reduction of discrimination threshold, the effect in the trained C55/S100 was indistinguishable from that in the untrained, surround-rotated, C55/S10 (p = 0.713; Fig. 2G, left blue and yellow bars), but it was significantly larger than that in the untrained, center-rotated, C145/S100 (p = 0.041; Fig. 2G, left blue and red bars). With respect to the reduction of perceptual bias, the effect was reversed: The reduction in the trained C55/S100 was significantly larger than that in the untrained, surround-rotated, C55/S10 (p = 0.029; Fig. 2H, left blue and yellow bars), but it was indistinguishable from that in the untrained, center-rotated, C145/S100 (p = 0.85; Fig. 2H, left blue and red bars). Figure 2I,J show the data from individual subjects contributing to the averaged results in Fig. 2G,H (left panels), respectively.

The above analyses implied that the improved discriminability was largely specific to the orientation of the task-relevant reference stimulus, and that the improved fidelity of orientation perception was specific to the orientation of the task-irrelevant contextual stimulus.

To further verify the above conclusion, after the mid-test (day 7), all of the subjects were trained for another five days (from day 8 to 12) in the orientation discrimination task using the two untrained stimulus configurations: C55/S10 and C145/S100 with altered surround and center orientations, respectively. For the surround-rotated C55/S10, the second training stage significantly decreased the perceptual bias (Fig. 2D: mid-test 2.90 ± 0.33° vs. post-test 1.81 ± 0.28°, n = 10, t = 3.54, p = 0.006, two-sided paired t-test), but the training had little effect on the discrimination threshold (Fig. 2C: mid-test 2.42 ± 0.19° vs. post-test 2.14 ± 0.28°, n = 10, t = 2.23, p = 0.053). Opposite effects were observed for the center-rotated C145/S100 in the second training stage: there was no significant change in the perceptual bias (Fig. 2F: mid-test 2.93 ± 0.46° vs. post-test 2.50 ± 0.31°, n = 10, t = 1.24, p = 0.25), but there was a significant reduction in the discrimination threshold (Fig. 2E: mid-test 3.11 ± 0.25° vs. post-test 2.31 ± 0.10°, n = 10, t = 2.89, p = 0.02). The differences between the mid- and post-tests in the orientation threshold and perceptual bias are summarized respectively in Fig. 2G,H (right panels). These data corroborated the conclusion that the decreased tilt illusion was specific to the task-irrelevant surround stimulus, whereas the enhanced orientation discriminability was specific to the task-relevant center stimulus.

Taken together, the results from both training stages in Experiment 2 consistently showed, in terms of orientation specificity, a dissociation between the enhanced discriminability and fidelity of orientation perception.

**Figure 3:**
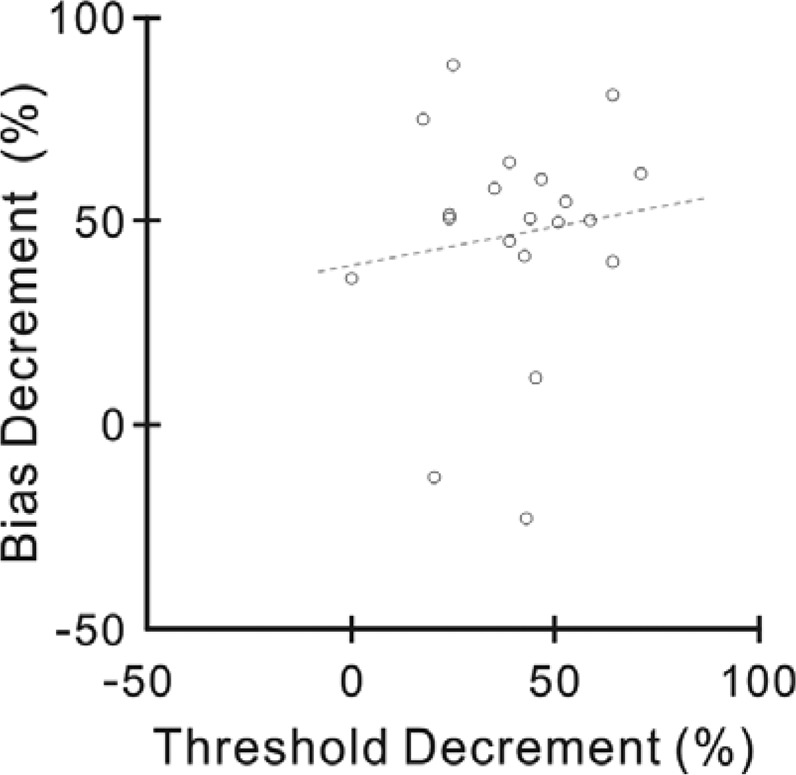
Comparison between percent changes in orientation discrimination threshold and perceptual bias across subjects. Different data points represent different subjects. The discrimination threshold data (and the corresponding estimates of perceptual bias) were pooled from the subjects trained on the C55/S10 in Experiment 1 (Fig. 1) and from those trained on the C55/S100 in Experiment 2 (Fig. 2). Dotted line shows the result of linear regression.

By pooling the subjects in Experiment 1 and 2, we calculated the percent decrement in the orientation threshold and perceptual bias for each subject. We found no significant correlation between the two practice effects across the subjects (Fig. 3C, Spearman correlation, n = 20, r = 0.074, p = 0.758). This analysis provided additional evidence for a dissociation between the two learning effects.

Further supporting evidence for a dissociation between the two learning effects came from the following experiments by manipulating the attended stimulus features during training.

### Increased fidelity of orientation perception dissociated from orientation discrimination training

Perceptual learning usually engages task-dependent top-down influences^27^; as a result, the learning effects usually do not generalize to task-irrelevant features of the stimulus used for training^28,29^. In Experiment 3 we examined whether the two learning effects observed in the current study were both restricted to training on orientation discrimination.

**Figure 4:**
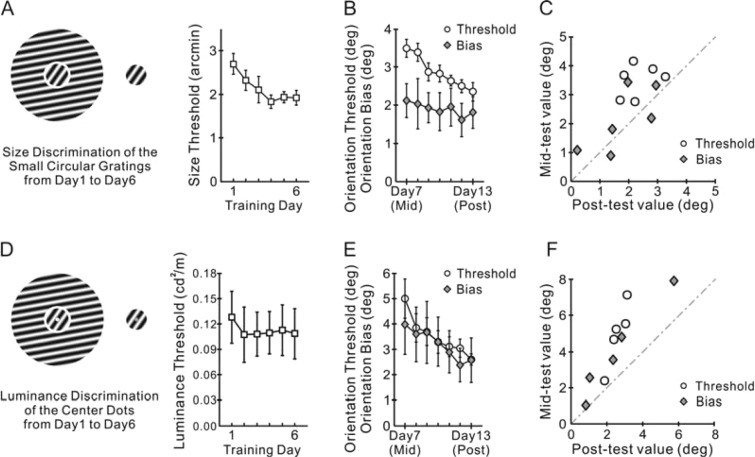
Effects of training in tasks irrelevant to orientation perception. In Experiment 3 (A-C, n = 6 subjects), after 6 days of training on the size discrimination task (**A**), subsequent 7 days of orientation discrimination training substantially reduced the orientation threshold but not the perceptual bias (**B**). Individual subjects’ orientation threshold and bias before (mid-test) and after (post-test) the orientation discrimination training are also shown (**C**). In Experiment 4 (**D**–**F**, n = 5 subjects), after 6 days of training on luminance discrimination of the central dots (**D**), subsequent 7 days of orientation discrimination training reduced both the orientation threshold and bias (**E**). Individual subjects’ orientation threshold and bias before (mid-test) and after (post-test) the luminance discrimination training are also shown (**F**). Error bars indicate ± SEM.

Six naive subjects were first trained to compare a difference in size between the reference and probe gratings (Fig. 4A; see Methods) from day 1 to 6, with all the other experimental settings kept identical to Experiment 1 (C55/S10). We did not measure the pre-training threshold and bias in orientation perception, because the learning effects induced by the pre-test of orientation discrimination would confound the measure of learning transfer induced by the size discrimination training per se. The averaged threshold for size discrimination was reduced from 2.70 ± 0.24 arc minutes in day 1 to 2.00 ± 0.17 arc minutes in day 6 (Fig. 4A; t = 2.62, p = 0.047, two-sided paired t-test), indicating that the subjects indeed paid attention to the size discrimination task.

To inspect whether the size discrimination training had any effect on orientation perception, the subjects subsequently practiced the orientation discrimination task from day 7 (mid-test) to day 13 (post-test). The training significantly reduced the orientation threshold (Fig. 4B, mid-test 3.49 ± 0.24° vs. post-test 2.35 ± 0.25°, n = 6, t = 4.19, p = 0.009, two-sided paired t-test). The amount of reduction was comparable to that observed in Experiment 1 (1.14 ± 0.27° in Experiment 3 vs. 1.94 ± 0.50° in Experiment 1, n_1_ = 6, n_2_ = 10, t = 1.26, p = 0.23, independent samples t-test), implying that the earlier size discrimination training had little effect on the orientation discriminability. In contrast, the bias in orientation perception was nearly unchanged during the subsequent orientation discrimination training (Fig. 4B, mid-test 2.12 ± 0.44° vs. post-test 1.81 ± 0.41°, n = 6, t = 0.95, p = 0.38, two-sided paired t-test). This amount of perceptual bias was comparable to that in the post-test of Experiment 1 (Fig. 1C), implicating that the bias had decreased to an asymptote by the previous size discrimination training. The data from individual subjects were rather consistent (Fig. 4C).

Experiment 3 showed that, unlike the improved orientation discriminability, the increased fidelity of orientation perception did not necessarily depend on training on orientation discrimination. Nevertheless, as demonstrated in the following Experiment 4, if the center-surround stimuli were entirely irrelevant to the trained task, the tilt illusion could not be reduced.

In Experiment 4, five naive subjects were instructed to ignore the grating patches by attending to the luminance difference between the two dots embedded respectively in the reference and probe grating patches (Fig. 4D; see Methods). The averaged threshold for luminance discrimination significantly decreased with the training (from 0.128 ± 0.03 cd/m2 to 0.108 ± 0.03 cd/m2; n = 5, t = 3.69, p = 0.021, two-sided paired t-test), indicating that the subjects indeed paid attention to the small dots.

After 6 days of training on discrimination of the dot luminance, the subjects performed the orientation discrimination task from day 7 (mid-test) to day 13 (post-test). Both orientation threshold and bias were significantly reduced after the orientation training (Fig. 4E; mid-test threshold 4.99 ± 0.77° vs. post-test threshold 2.60 ± 0.23°, n = 5, t = 4.25, p = 0.01; mid-test bias 3.97 ± 1.17° vs. post-test bias 2.57 ± 0.87°, n = 5, t = 3.92, p = 0.02; see also individual subjects’ data in Fig. 4F).

Experiment 4 showed that a training task entirely irrelevant to the center-surround gratings had little effect on orientation perception. This result, together with those from Experiment 1–3, also implicated an important role of selective attention to the center stimulus in learning to improve both perceptual discriminability and fidelity.

## Discussion

We have studied orientation discrimination of target stimuli subjected to a context-induced tilt illusion. We demonstrated that orientation discrimination training improved not only the orientation discrimination ability, but also the fidelity of orientation perception, by rendering the subjective perception less susceptible to the contextual influences. The two learning effects showed differential specificities to the stimulus settings used for training. The improved fidelity of orientation perception was specific to the task-irrelevant surround stimulus but not specific to the task-relevant center stimulus. This learning effect on the perceptual fidelity was even independent of the tasks performed on the center stimulus as long as one of its features (e.g. the size) was used for training. In contrast, the improved orientation discriminability was specific to the task-relevant center stimulus but not specific to the task-irrelevant surround. Our observations indicate that the same training procedure can generate two dissociable learning effects. This conclusion was further supported by the observation that the sizes of the two learning effects were uncorrelated across the subjects. Our findings suggest that the two practice effects may engage two parallel learning processes: a task-dependent process giving rise to enhanced discriminability for the task-relevant stimulus attribute, and a context-dependent process leading to improved perceptual fidelity for the attended stimuli.

### Comparisons with previous studies

Previous studies have reported that an attentive inspection of illusory stimuli can temporarily reduce the illusion^30–32^. This can be well explained as a result of short-term adaptation or dynamic adjustment of attentional deployment, but these factors cannot account for the long-lasting (> 4 months) decrement of the tilt illusion associated with perceptual learning.

A previous study trained human and monkey observers in a brightness discrimination task in the presence of context-induced bias in brightness perception^21^. The authors reported a concurrent decrease in brightness threshold and perceptual bias, but the two learning effects were evident only when the observers were required to distribute attention to multiple stimuli, in contrast with the negligible effects under focal attention. An adjustment of attentional deployment, from distributed to focal attention, was used to account for the two learning effects that were characterized by a lack of specificity to stimulus orientation and location. Such an account cannot be applied to the observations in our current study: Even if the observers learned to adjust their attentional deployment, this effect of attentional learning could not explain the two learning effects that showed dissociable orientation specificities and were uncorrelated in magnitudes across subjects.

Several studies on perceptual learning, in the absence of any context-induced illusion, reported mixed results in terms of post-training effects on perceptual fidelity. A recent study trained human observers to discriminate the direction of moving dots^33^. After the training was completed, a context of moving dots was added, producing a repulsive effect on the perceived motion direction. The authors found that the illusion was significantly weaker in comparison with the illusion before the training. This post-training effect, which was specific to the direction discrimination task and to the trained motion direction, was thought to share the same mechanisms underlying improved discriminability. Another study trained human subjects to discriminate the orientations of an isolated grating patch without contextual stimuli^23^. A significant post-training bias (impaired perceptual fidelity) in orientation perception was observed ~15° away from the trained orientation, which could be explained by learning- or adaption-induced changes in orientation tuning functions of V1 neurons around the trained orientation^25^ (for more details see the following discussion on **Speculated mechanisms of the dissociable learning effects**). Although the above studies reported seemingly contradictory results in terms of changes in perceptual bias after perceptual learning in the absence of visual illusions, they all argued for a shared mechanism between the improved discriminability and the post-training changes in perceptual bias.

A modeling study has postulated that perceptual bias and discriminability follow a simple mathematical relation when they are quantified as functions of the stimulus parameters such as orientation^34^. However, such a simple mathematical relation does not necessarily implicate that the two perceptual functions and their learning processes are inherently linked. This point of view is supported by a study showing that, in spite of a significant improvement in orientation discriminability, the tilt illusion remains unaltered at the orientation previously used for orientation discrimination training^24^. Our findings also support this point of view by showing two dissociable learning effects. For example, the size discrimination training did not affect the orientation discrimination threshold but it decreased the tilt illusion (Fig. 4). A critical difference between our current study and previous perceptual learning studies (except for ref. ^21^) is that we trained subjects in the presence of context-induced illusion. Our findings support that not only the two perceptual abilities per se but also their improvements with training are dissociable.

### Speculated mechanisms of the dissociable learning effects

The neural mechanisms of perceptual learning have been extensively discussed in many review articles from different perspectives (e.g. refs. ^1,2,22,35–37^). Here we mainly focus on learning-induced changes in perceptual fidelity that has been largely overlooked. We speculate that the mechanisms responsible for the reduced tilt illusion could be different from those for improved discriminabilities.

It has been proposed that orientation perception is mediated by the concerted activities of a population of orientation selective neurons^38,39^. A change in the population code by training could result in altered discriminability and/or fidelity of orientation perception. Changes in neuronal response property associated with orientation discrimination training have been reported in monkey V1^40^ and V4^41,42^ (but see ref. ^43^ for a different point of view supporting rule-based learning in higher-order areas). In particular, a neural correlate of improved orientation discriminability—the sharpening of V1 orientation tuning curves in the trained retinotopic region^40^—is well explained by a recurrent model of V1 orientation selectivity^25^. This model also predicts a shift in the vector sum of V1 population responses (in the absence of contextual influences), but such a shift takes place only at untrained orientations. Therefore, this postulated mechanism underlying improved orientation discriminability cannot explain the reduction of the tilt illusion at the trained orientation observed in the current study.

The tilt illusion itself has been attributed to contextual influences mediated by the lateral interactions between orientation-selective neurons^16,44^. The contextual influences can dynamically modify neurons’ orientation tuning curves, causing a shift in the vector sum of neuronal population responses^16,45^. The size of the shift, and therefore, the size of the tilt illusion would be dependent on the strength of the lateral interactions between neurons, as supported by human imaging studies:^46,47^ The strength of effective connectivity between two retinotopic regions in V1 activated by the center and surround stimuli is correlated with the magnitude of the tilt illusion.

We speculate that, in the current study, the learning-induced reduction of the tilt illusion could be due to a weakening of the lateral interactions or effective connectivities between neurons encoding the center and surround stimuli. Contextual interactions seen in V1 can be modified by top-down influences and by perceptual training (for reviews see ref. ^2,36^). It has been shown that top-down feedback modulations play an important role in dynamically gating or adjusting the effective connectivity of horizontal connections ^15,48^. Extended training under repeated top-down influences can even modify the axon collaterals forming the horizontal connections^49^. It has been proposed that cortical plasticity associated with perceptual learning engages two types of modulatory signals: reward-related diffusive neuromodulatory signals (such as dopamine and acetylcholine) that are responsible for synaptic changes, and top-down selection signals for guiding the synaptic plasticity^35,37^. However, a puzzling question calls for further investigations: if extended training in a specific task could cause a relatively permanent modification of the anatomical connections, would such a change interfere with the processing of untrained stimuli and tasks?

Besides the changes in neural effective connectivity mediating the center-surround interactions, the decreased contextual influences could also be accounted for by an increase in suppression of neural activities to the contextual stimuli, probably through top-down attentional influences and/or long-term visual adaptations. It has been shown that attentional modulation of visual cortical responses not only enhances the representation of task-relevant stimulus but also suppresses irrelevant distractors^50–54^. Moreover, training can further enhance attentional suppression of task-irrelevant or interfering stimuli, and such suppressive learning effects show a certain degree of specificity to the contextual stimuli^19,20^. In addition to learning to suppress task-irrelevant stimulus context (an active or effortful process), a form of long-term (cumulative) passive adaptation or habituation could take place in early visual cortex due to long-term repeated stimulation of the same task-irrelevant stimuli^55^. It is plausible that the rate of adaptation or habituation to the task-irrelevant surround stimulus (which may reflect local visual cortical properties) and the rate of attention-driven learning for the task-relevant attribute of the center stimulus (which may rely on recurrent processes involving a network of cortical areas) would be uncorrelated across individuals. These two learning processes might form the basis of the individually uncorrelated learning effects of reduced perceptual bias and enhanced discrimination ability. The specificity of adaptation effects could be in line with the specificity of training-induced reduction of the tilt illusion. On the other hand, a pure passive adaptation effect could not explain the task and stimulus contingencies observed in the current study, so some interactions between attention and adaptation might be involved.

Our current study indicates that the behavioral consequences of perceptual training could be multifaceted even for a simple perceptual task, consistent with previous studies showing complex interactions among attention, contextual influences, and learning. Our findings also support the notion that perceptual training affects multiple aspects of visual perception^56,57^ and modifies multiple neural processes across cortical areas^2,22^.

## Data Availability

Data are available from the corresponding author on reasonable request.
